# Body Mass Index Measured Repeatedly over 42 Years as a Risk Factor for Ischemic Stroke: The HUNT Study

**DOI:** 10.3390/nu15051232

**Published:** 2023-02-28

**Authors:** Jens W. Horn, Tingting Feng, Bjørn Mørkedal, Dagfinn Aune, Linn Beate Strand, Julie Horn, Kenneth J. Mukamal, Imre Janszky

**Affiliations:** 1 Department of Public Health and Nursing, Norwegian University of Science and Technology, 7034 Trondheim, Norway; 2 Department of Internal Medicine, Levanger Hospital, Health Trust Nord-Trøndelag, 7600 Levanger, Norway; 3 Department of Health Register, The Norwegian Directorate of Health, 0213 Trondheim, Norway; 4 Department of Cardiology, Oslo University Hospital, Rikshospitalet, 0372 Oslo, Norway; 5 Department of Epidemiology and Biostatistics, School of Public Health, Imperial College London, St. Mary’s Campus, Norfolk Place, Paddington, London W2 1PG, UK; 6 Department of Nutrition, Oslo New University College, 0456 Oslo, Norway; 7 Department of Endocrinology, Morbid Obesity and Preventive Medicine, Oslo University Hospital, 0372 Oslo, Norway; 8 Department of Obstetrics and Gynecology, Levanger Hospital, Nord-Trøndelag Hospital Trust, 7600 Levanger, Norway; 9 Department of Medicine, Beth Israel Deaconess Medical Center, Boston, MA 02215, USA; 10 Department of Global Public Health, Karolinska Institutet, 17 176 Stockholm, Sweden; 11 Regional Center for Health Care Improvement, St. Olav’s University Hospital, 7030 Trondheim, Norway

**Keywords:** body mass index, BMI, overweight, obesity, ischemic stroke, epidemiology, population-based cohort study

## Abstract

Background: Higher BMI in middle age is associated with ischemic stroke, but little is known about BMI over adulthood, and the risk for ischemic stroke as most studies relied on a single measurement of BMI. Methods: BMI was measured four times over a period of 42 years. We calculated average BMI values and group-based trajectory models and related these to the prospective risk of ischemic stroke after the last examination in Cox models with a follow-up time of 12 years. Results: A total of 14,139 participants, with a mean age of 65.2 years and 55.4% women, had information on BMI from all four examinations, and we observed 856 ischemic strokes. People with overweight and obesity over adulthood had a higher risk for ischemic stroke with a multivariable-adjusted hazard ratio of 1.29 (95% CI 1.11−1.48) and 1.27 (95% CI 0.96−1.67), respectively, when compared to normal weight participants. Excess weight tended to have stronger effects earlier than later in life. A trajectory of developing obesity throughout life was associated with higher risk than other trajectories. Conclusions: High average BMI, especially at an early age, is a risk factor for ischemic stroke. Early weight control and long-term weight reduction for those with high BMI may decrease the later occurrence of ischemic stroke.

## 1. Introduction

The age-standardized incidence and mortality rate of ischemic stroke have declined during the last several decades due to improved prevention and therapy [1,2,3]. Nonetheless, ischemic stroke is still the second most frequent cause of death [4] and the second most common cause of disability worldwide [5]. Due to the increasing age of the global population, the total number of ischemic strokes is projected to increase in the future [4]. Identification and treatment of modifiable risk factors are therefore important to improve primary prevention. While high income countries have shown impressive reductions in blood pressure and cholesterol levels, the intertwined epidemics of diabetes mellitus, overweight and obesity have worsened [2]. The prevalence of people with obesity is 14–32% in different European countries and 34% in the USA [6], and it is the fastest-growing risk factor for stroke worldwide [4,7]. Previous studies have suggested that body mass index (BMI) has a dose-dependent, J-shaped association with the risk for ischemic stroke [2,8,9]. The great majority of these studies relied on a single measurement of BMI, despite dynamic changes in body weight over the lifetime [10]. Few studies have examined repeated assessments of BMI in relation to ischemic stroke risk over decades, often using self-reported body weights, and their results are conflicting [3,10,11]. Self-reported current body weight and height can be accurate, however, recalled information on body weight, especially from decades earlier, is prone to misclassification, and its accuracy depends on several characteristics, including the actual values of body weight. Thus, it may cause considerable bias [12].

This study was the first to examine the prospective associations between BMI measured repeatedly over four decades in adulthood and the risk of ischemic stroke in a large population-based study.

## 2. Materials and Methods

### 2.1. Study Population

The Trøndelag Health Study (HUNT) is an ongoing longitudinal population-based study with several waves to which all inhabitants 20 years and older from North Trøndelag county were invited. The relevant waves for this work were HUNT1, conducted in 1984–1986, HUNT2, conducted in 1995–1997, and HUNT3, conducted in 2006–2008. Apart from these three phases of the HUNT study, mandatory tuberculosis screening program where height and weight were measured was conducted in the region between 1966 and 1969 that included all inhabitants older than 15 years of age [13]. In HUNT3, which was regarded as the baseline for the present study, 93,860 residents were invited to participate. Of those invited, 54% (50,801) answered the questionnaires and underwent clinical examinations [14,15]. However, for the most relevant age groups, i.e., among those with high risk for stroke, this number was much higher: 71.1% and 66.8% for those 60–69 and 70–79 years old, respectively [15]. Non-participation studies showed that the main reasons for non-participation in HUNT were “being very busy, forgetting the invitation or not being interested” and that non-participants had somewhat lower socioeconomic status and higher mortality than participants [16].

Height and weight were measured in 48,871 HUNT3 participants (96%). We excluded 1532 participants with a diagnosis of ischemic stroke (self-reported or indicated in hospital records) prior to study entry (i.e., baseline measurement at HUNT3). Of the HUNT3 participants who did not have an ischemic stroke before baseline and who had an available BMI at baseline, 15,348 participants had information on BMI from all previous measurements, i.e., the tuberculosis screening, HUNT1 and HUNT2. Out of these, we excluded 1209 participants due to a lack of baseline information on covariates (marital and smoking status, alcohol consumption, physical activity and education), and accordingly, 14,139 participants were included in the final sample for the main analysis (Figure S1).

### 2.2. Exposure Assessment

Weight and height measurement, calculation of average BMI and weight change.

At each of the four assessments, weight and height were measured to the nearest centimeter or nearest half kilogram while participants were wearing light clothes without shoes. BMI was calculated as body weight in kilograms divided by the square of height in meters and categorized into four groups consistent with World Health Organization cut points: <18.5 kg/m^2^ (underweight), 18.5–24.9 kg/m^2^ (normal weight), 25–29.9 kg/m^2^ (overweight) and ≥30 kg/m^2^ (obese). Body weight and height measurements for individuals who were adolescents at the time of the tuberculosis survey were converted to adult BMI categories using internationally recognized cut points [17,18].

**Figure 1:**
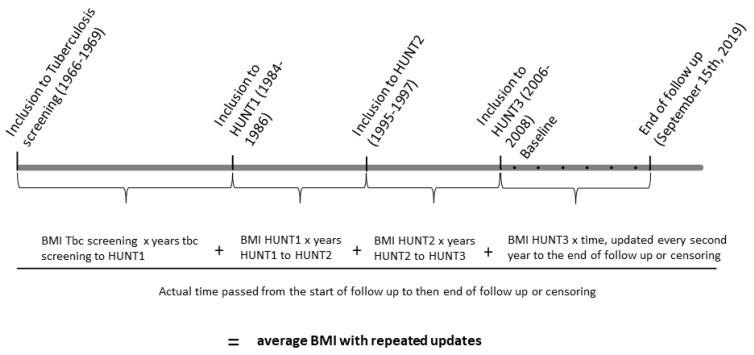
Graphic Presentation of the Study Setup and the Calculation of Average BMI.

We denoted the BMI values at the four time points here as BMI_67_ (obtained from the tuberculosis screening program conducted in 1966–1969), BMI_85_ (HUNT1 conducted in 1984–86), BMI_96_ (HUNT2 conducted in 1995–1997) and BMI_07_ (HUNT3 conducted in 2006–2008). We estimated the average BMI from BMI_67_ to the end of the follow-up period by taking the different follow-up times due to censoring into account. Therefore, we updated the time and the average BMI after baseline every other year (See the graphical presentation of the study setup and the formula in Figure 1). By doing so, the length of the follow-up period had less influence on the average BMI, and censored participants became more comparable to the uncensored participants.

The equation for calculating average BMI from BMI_67_ to the end of follow up by repeated updated exposure time after baseline was:

Average BMI_67-EOF_ = ((BMI_67_ × time_I-II_) + (BMI_85_ × time_II-III_) + (BMI_96_ × time_III-IV_) + (BMI_07_ × time_IV_-time_EOF_))/time_I-EOF_

where:

time_I-II_ = time from measurement I (i.e., in 1966–1969) to measurement II (i.e., in 1984–1986).

time_II-III_ = time from measurement II to measurement III (i.e., in 1995–1997).

time_III-IV_ = time from measurement III to measurement IV (i.e., 2006–2008).

time_IV-EOF_ = time from measurement IV to the end of follow-up (i.e., 2006–15 September 2019, updated every other year).

time_I-EOF_ = time from measurement I to the end of follow-up (updated every other year).

When the average BMI includes the BMI from HUNT3, we have highly correlated independent variables in our statistical models. Therefore, to address multicollinearity in the analysis of whether past or recent BMI was a more important risk factor of stroke, we also calculated average BMI only prior to baseline (HUNT3 2006–2008):

Average BMI_67-0_ = ((BMI_67_ × time_I-II_) + (BMI_85_ × time_II-III_) + (BMI_96_ × time_III-IV_))/time_I-IV_

where:

time_I-IV_= time from measurement I (i.e., in 1966–1969) to measurement IV (i.e., in 2006–2008).

We calculated the total BMI change from BMI_67_ to BMI_07_ by the following equation:

Total BMI change_67-07_ = (((BMI_85_ − BMI_67_) × time_I-II_) + ((BMI_96_ − BMI_85_) × time_II-III_) + (BMI_07_ − BMI_96_) × time_III-IV_))/time_I-IV_

After calculating “Total BMI change” (BMI_07_ − BMI_67_), we calculated BMI change in the “Early period” (BMI_85_ − BMI_67_), the “Middle period” (BMI_96_ − BMI_85_) and in the “Late period” (BMI_07_ − BMI_96_). We classified these BMI changes in 3 categories: <−2.5 kg/m^2^, ≥−2.5 to <2.5 kg/m^2^ and ≥2.5 kg/m^2^.

We used Stata Traj Plugin for identifying group-based trajectories. [19]. More detailed information about group-based trajectory modeling is provided in the Supplemental Material (File S1, Tables S5–S7).

### 2.3. Outcome Assessment

Ischemic stroke diagnoses were retrieved from the electronic patient administrative systems of all hospitals providing acute medical care in Trøndelag (Levanger Hospital, Namsos Hospital and St. Olav`s Trondheim University Hospital). We used the International Classification of Disease (ICD) Ninth Revision code 433 and 434, and ICD Tenth Revision code I.63 to identify ischemic strokes from September 1987 (after the start of the electronic administrative system) to 15 September 2019 (timepoint of data extraction). The ICD codes for ischemic stroke have been found to be of high validity within the HUNT cohort [20,21].

### 2.4. Covariates

Information on covariates was retrieved from the HUNT3 questionnaires or clinical examination. Participants self-reported their marital status (unmarried, married/cohabitant, widowed/divorced/separated), smoking status (never, former, occasional, current), and alcohol consumption (abstainer, light drinker, moderate drinker and heavy drinker based on the self-reported type, amount and frequency of consumption of different alcoholic beverages). Participants reporting ≥3 h of light exercise or ≥ one hour of hard exercise per week were regarded as physically active. Based on self-reported occupation, we derived educational level according to the International Standard Classification of Occupation from Statistics Norway and categorized it into: “lower secondary school level” (10 years of schooling or less), “higher secondary school level” (10–12 years of schooling) and “tertiary education” (any fulfilled education at university or college) [22]. For 690 participants with missing information on occupation in HUNT3, we retrieved information on educational level from HUNT2 questionnaires. We also calculated the number of self-reported common chronic diseases from the following list: kidney diseases, chronic obstructive lung disease, heart failure, asthma, obstructive sleep apnea, psoriasis, cancer, epilepsy, rheumatoid arthritis, ankylosing spondylitis, sarcoidosis, osteoporosis, osteoarthritis, fibromyalgia, chronic physical pain, hyper- and hypothyroidism.

All physical examinations were performed at local field centers by trained staff. Blood pressure was measured three times in a sitting position after a short rest with a Dinamap Critikon model 8100. Hypertension was defined as systolic blood pressure >130 mmHg or diastolic blood pressure >90 mmHg or as self-reported use of blood pressure medication. Non-fasting blood samples were analyzed for glucose, triglycerides and high-density lipoprotein cholesterol (HDL-C). Diabetes was defined based on self-report or on non-fasting serum glucose >11.1 mmol/L.

### 2.5. Statistical Analysis

Baseline characteristics were presented for continuous variables as mean ± standard deviation (SD) and for categorical ones as percentages.

For the main analysis, we used Cox proportional hazard models where age was the underlying time scale to calculate hazard ratios (HRs) with 95% confidence intervals (CIs) for first-ever ischemic stroke for given categories of average BMI, with normal weight being the reference group. Participants contributed person-time from the date of HUNT3 participation until the date of first stroke diagnosis, date of death, emigration, or end of follow-up (15 September 2019), whichever occurred first. To calculate the average BMI to the end of follow-up, we updated the time since baseline every second year by the "stsplit" function in STATA. The proportional hazard assumption for each covariate was tested by -ln-ln survival curves and Schoenfeld residuals. We found no violations of the proportional assumption.

Confounders were chosen by a priori knowledge about their relationships to the exposure and outcome. All models were adjusted for age and sex (model 1). In model 2, we further adjusted for smoking status, education, marital status, physical activity, alcohol consumption, and common chronic diseases. In a further model, we additionally adjusted for potential mediators such as hypertension, diabetes, HDL-C and triglycerides. To address the relative importance of the recent and past BMI in predicting ischemic stroke risk, we also adjusted for BMI at HUNT3 (baseline).

In a sensitivity analysis, we stratified for sex and age at HUNT3 (<65/≥65 years) or smoking (never/former, current or occasionally smoker).

We identified three groups based on the trajectories of BMI development, and these groups were also included in our Cox models with consistent normal weight as the reference group.

In another analysis, we included a change in BMI over the entire follow-up period and in discrete time periods in the Cox models. These analyses were adjusted for confounders from model 2 and the regression slope of BMI from BMI_67_ to BMI_07_ to account for increasing BMI over time. Change in BMI was categorized into three groups, where the group with no essential change of BMI (≥−2.5 to <2.5 kg/m^2^) was the reference.

We performed cubic splines for a graphical presentation of the hazard ratios for ischemic stroke in relation to BMI_07_ (i.e., measured at HUNT3) and average BMI from tuberculosis screening to the end of follow-up with 4 knots. For cubic splines of BMI at HUNT3, we adjusted for confounders from model 2 and, additionally, in a separate model, for the average BMI from tuberculosis screening to HUNT3. For cubic splines of the average BMI, we adjusted for covariates in model 2 and additionally for BMI at HUNT3.

All statistical analysis was conducted using Stata 17 for Windows (StataCorp LP, College Station, TX, USA). The codes are available from the corresponding author upon reasonable request.

The study was approved by the regional committee for ethics in medical research (2016/542REC Central), the National Directorate of Health, and the Norwegian Data Inspectorate. All HUNT study participants provided informed consent.

We followed the STROBE guideline for prospective cohort studies.

## 3. Results

Of 14,139 participants at the HUNT3 visit, 55.4% were female, the mean age was 65.2 years (SD 9.3 years), 15.4% were current smokers, and 20.1% were physically inactive. Mean BMI increased progressively over time from 23.2 kg/m^2^ (SD 3.1) at tuberculosis screening program to 27.6 kg/m^2^ (SD 4.2) at HUNT3 (Table 1). The excluded population primarily lacked information on BMI from the tuberculosis screening (86.5%) and was younger and consequently had a lower blood pressure, lower prevalence of diabetes and smoking, a more favorable lipid profile, and better education. During the follow-up period of 12 years and 152,843 person-years, a total of 856 participants suffered a first ischemic stroke.

### 3.1. Body Mass Index and Risk for Ischemic Stroke

With both approaches to calculating average BMI, the risk of ischemic stroke increased with increasing average BMI (Table 2). The HRs were comparable, but the estimates were slightly higher when we calculated average BMI to the baseline. When we adjusted for BMI at HUNT3, the estimates for overweight and obesity were somewhat attenuated when the average BMI was calculated to the end of the follow-up period but was nearly unchanged when the average BMI was calculated to the baseline. Adjustment for metabolic consequences of obesity also attenuated the association between obesity and ischemic stroke risk (Table S1).

**Figure 2:**
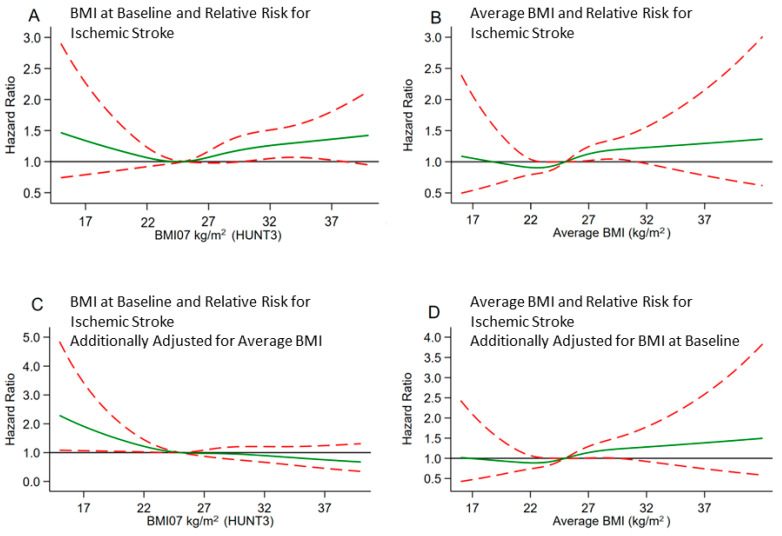
(**A** to **D**): Cubic Splines of BMI at Baseline or Average BMI (BMI_67-07_) and HR of Ischemic Stroke. All Splines are Adjusted for Covariates of Model 2. (**C**) is Additionally Adjusted for Average BMI (BMI_67-07_) and (**D**) for the Most Recent BMI from Baseline HUNT3.

Figure 2A shows the association of the most recent BMI, i.e., measured at HUNT 3, with risk for ischemic stroke. The risk increased steadily with BMI values higher than 27 kg/m^2^. When we adjusted for the average BMI until the last measurement at HUNT3, the elevated risk with higher BMI values disappeared (Figure 2C). Using average BMI from tuberculosis screening to the end of follow-up, we observed a similar association as when we used BMI from HUNT3 (Figure 2B). Adjustment for BMI from HUNT3 had little effect on the association (Figure 2D). When we examined the risk associated with BMI from each period of assessment separately, the multivariable-adjusted HRs were highest for obese and overweight participants at the tuberculosis screening and HUNT1 and attenuated gradually for both BMI groups at later HUNT examinations (Table S4).

**Figure 3:**
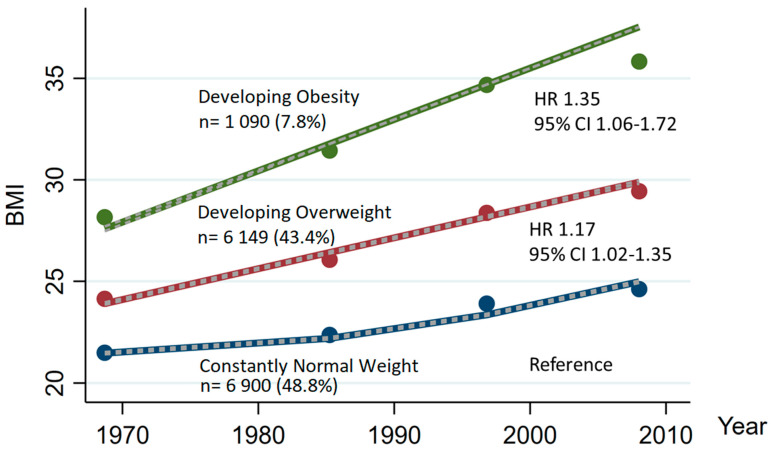
Group Based Trajectories of BMI over 42 years and Risk for Ischemic Stroke. HR Adjusted for Covariates of Model 2.

We identified three distinct BMI trajectories based on group-based trajectory modeling and referred to these groups as consistently normal weight, developing overweight and developing obesity, representing 48.8% (n = 6900, events= 345), 43.4% (n = 6149, events = 425) and 7.8% (n = 1090, events = 86) of the participants, respectively (Figure 3). In all these categories, the BMI increased over time. The participants in the "developing obesity group” had higher blood pressure, higher prevalence of diabetes, a slightly less favorable lipid profile and reported less physical activity compared to participants in the two other groups (Table S2). The multivariable-adjusted HR for ischemic stroke was higher for the developing overweight and developing obesity group compared to the consistently normal weight group, an association that was attenuated when adjusting for established metabolic consequences of obesity. Additional adjustments for the most recent BMI had no effect on the estimates.

High BMI tended to have a stronger association with stroke among men than among women. In contrast, age and smoking status did not modify the association with the risk of ischemic stroke (Table S3).

### 3.2. Change in Body Mass Index and Risk for Ischemic Stroke

When we examined BMI changes over the whole period and at early, middle and late periods separately, we found 34% higher risk (95% CI 3–75%) of ischemic stroke for weight loss in the late period. Other weight changes were not associated with risk for ischemic stroke, although in some of the categories, our statistical power was limited (Table S4).

## 4. Discussion

In this large population-based study, we observed a higher risk for ischemic stroke among participants with obesity (BMI >30 kg/m^2^) or overweight (BMI 25–30 kg/m^2^) over adulthood. The associations of excess weight with risk were particularly evident for obesity earlier rather than later in life. In addition, trajectory analysis found that developing overweight or obesity over time was associated with higher risk.

Our findings regarding body weight at baseline and risk of subsequent ischemic stroke are generally in line with previous studies, although the association was somewhat weaker in the present study than in previous ones [2,8,9]. Compared to normal weight participants, obese participants at baseline were at 17% higher risk for ischemic stroke in our comprehensive multivariable-adjusted model. In a meta-analysis, however, the corresponding pooled estimate was 1.64 (95% CI 1.36–1.99) in the unadjusted model, and a subgroup analysis of multivariable-adjusted studies had a pooled risk ratio of 1.50 (95% CI 1.34–1.67) [9]. There are several differences between our study and this large meta-analysis of studies, including various races and applying different statistical adjustments. Most importantly, our study population was considerably older than participants in most of the studies included in the meta-analysis. The relative risk for cardiovascular disorders can markedly decrease with increasing age [23], and paradoxically overweight can even have an inverse association at very old ages [24].

Our study uniquely included anthropometric information based on actual measurements performed decades before baseline. Previous studies that incorporated long-term information relied partly on self-reported BMI [3,10,11]. However, recalled BMI is potentially biased as its accuracy depends on actual body weight in the past [12]. In this study, a high average BMI was associated with increased ischemic stroke risk, and this association was not explained by having a high BMI at baseline (HUNT3) since adjusting for the most recent BMI from HUNT3 virtually had no effect on the estimates. In contrast, the association with BMI from HUNT3 was essentially eliminated by adjustment for earlier BMI. Our findings thus support the significance of early adulthood weight gain and the long-time exposure to excess fat tissue as a risk factor for ischemic stroke. To the best of our knowledge, no previous studies have compared the relative importance of earlier and current excess weight and risk of ischemic stroke.

When analyzing BMI trajectories, all trajectory groups showed increasing weight over time, compared with earlier studies [10,25]. The "developing obesity" group had the highest risk for ischemic stroke. Earlier studies using recalled BMI generally came to the same conclusion [3,10]. Of note, trajectory analysis suggested the presence of three predominant trajectories, which effectively represented stable degrees of normal weight, overweight, or obesity. This observation tends to align with the relative lack of power observed for most categories of weight change.

In line with most previous studies, we observed a higher risk for ischemic stroke among participants in middle age with recent weight loss but no association with recent weight gain [3,26,27]. Similarly to other observational studies, we could not differentiate between voluntary and involuntary weight loss. Consequently, the adverse association of weight loss with stroke risk might well reflect confounding by comorbidities leading to both weight loss and elevated stroke risk. Studies on dramatic voluntary weight loss, i.e., after bariatric surgery, documented clear clinical benefits regarding cardiovascular diseases, hypertension, hyperlipidemia, and diabetes [28].

Ischemic stroke is typically due to large and/or small vessel diseases or due to cardio-embolic events. Excess weight can affect these different etiologies via several mechanisms, including hypertension, diabetes mellitus, low HDL-C and increased triglycerides [2], which can, to different degrees, increase the risk of the different ischemic stroke subtypes [29]. Both long-lasting and short-term excess of body fat are important risk factors for diabetes mellitus [10], but only long-lasting excess body fat is associated with the development of hypertension and hypercholesterolemia. Since hypertension is the single most important risk factor for ischemic stroke, the strong association of hypertension with lifelong adiposity might explain the differential importance of current and earlier excess weight observed in the present study [30,31].

Apart from these well-known metabolic consequences, additional pathways may mediate the effect of increased fat tissue on the risk of an ischemic stroke. Fat tissue, especially visceral, is endocrinologically active and may contribute to increased systemic inflammation and reduce the excretion of adiponectin, which in turn can also facilitate insulin resistance, arteriosclerosis and endothelial dysfunction [32]. Atrial fibrillation, a major cause of cardio-embolic stroke, is also known to be influenced by long-term obesity partly via increased pericardial fat [33,34]. Obesity increases the risk of obstructive sleep apnea, which can increase the risk of ischemic stroke. The pathophysiological pathways are complex, and besides increased blood pressure and sympathetic activation, endothelial, inflammatory, metabolic and other mechanisms are also likely to be involved [35].

### Strength and Limitations

Nord-Trøndelag county has a homogenous and stable population with a migration of a mere 0.3% per year. We had an exceptionally long exposure window before baseline to capture the development of body weight. We also had virtually complete follow-up utilizing validated local and nationwide registers of diagnoses of stroke [20,21,36]. Furthermore, we included a wide range of covariates in this highly-phenotyped population.

Despite its strengths, our study also had some limitations. BMI does not differentiate between fat and muscle tissues. [37,38]. Due to a low number of cases, our precision was very low in some BMI groups, as reflected by the wide confidence intervals. We had no information about the use of antiplatelets, anticoagulants or lipid-lowering medications, although these are unlikely to confound our results considerably as we extensively controlled for demographic and lifestyle factors which are associated with the use of these medications. Moreover, as we emphasized above, we had no information on whether the weight loss of the participants was intentional. This makes our results on weight loss, especially on late weight loss, difficult to interpret, as many diseases can cause weight loss and might also increase the risk of ischemic stroke. We also had limited statistical power to investigate stroke risk among underweight participants as the number of participants and events was low in this group. Finally, our findings in this Norwegian cohort are not directly generalizable to other populations.

## 5. Conclusions

High average BMI, especially at an early age, was a risk factor for ischemic stroke. The association persisted even after adjustment for the most recent BMI, suggesting cumulative effects over time. Early weight control and long-term weight reduction for those with high BMI may decrease the occurrence of stroke.

## Figures and Tables

**Table 1:** Characteristics of the Study Population (n = 14,139).

Age at HUNT3 (years)	65.2 (9.3)
Female, n (%)	7838 (55.4)
BMI_67_ (kg/m^2^)	23.2 (3.1)
BMI_85_ (kg/m^2^)	24.7 (3.3)
BMI_96_ (kg/m^2^)	26.7 (3.8)
BMI_07_ (kg/m^2^)	27.6 (4.2)
SBP (mmHg) missing n = 40, (0.3%)	137.5 (19.4)
DBP (mmHg) missing n = 40, (0.3%)	75.3 (11.4)
Hypertension, n (%)	10,550 (75.2)
Diabetes mellitus, n (%)	1018 (7.2)
Total cholesterol (mmol/L) missing n = 318 (2.2%)	5.76 (1.12)
HDL cholesterol (mmol/L) missing n = 318 (2.2%)	1.38 (0.37)
Triglycerides (mmol/L) missing n= 141 (1%)	1.71 (0.95)
Smoking status:	
Never, n (%)	5528 (39.1)
Former, n (%)	5681 (40.2)
Occasionally, n (%)	748 (5.3)
Current, n (%)	2182 (15.4)
Alcohol consumption:	
Abstainers, n (%)	3526 (24.9)
Light drinkers, n (%)	7810 (55.2)
Moderate drinkers, n (%)	2589 (18.3)
Heavy drinkers, n (%)	214 (1.5)
Education:	
Lower secondary, n (%)	6242 (44.2)
Upper secondary, n (%)	5637 (40.0)
Tertiary, n (%)	2260 (16.0)
Physical inactivity	
Inactive, n (%)	2840 (20.1)
Active, n (%)	11,299 (79.9)
Marital Status:	
Single, n (%)	782 (5.5)
Married, Cohabitant, n (%)	9962 (70.5)
Widow, Divorced, Separated, n (%)	3395 (24.0)
Participants with number of chronic diseases:	
No, n (%)	7010 (49.6)
One, n (%)	2810 (19.9)
Two, n (%)	2159 (15.3)
Three, n (%)	1214 (8.6)
Four, n (%)	572 (4.0)
Fife or more, n (%)	374 (2.6)

Values are presented as mean ± standard deviation or number (percentages). BMI body mass index, SBP systolic blood pressure, DBP diastolic blood pressure, HDL high-density lipoprotein.

**Table 2:** Hazard Ratios (HRs) of Ischemic Stroke, by Categories of Average Body Mass Index to the end of Follow-up with Repeated Updated Exposure and by Categories of Average Body Mass Index Until the HUNT3 Measurement (BMI_07_).

Mean BMI (kg/m^2^)	N 14,139	Events 856	Person Years 152,843	HR ^§^	95% CI	HR ^‖^	95% CI	HR ^#^	95% CI
Average BMI from BMI_67_ to the end of follow up with repeated updated exposure									
<18.5	36	1	405	0.60	0.08–4.24	0.55	0.08–3.90	0.57	0.08–4.09
18.5–<25	7182	363	78,735	1	(Ref)	1	(Ref)	1	(Ref)
25–29.9	5895	420	62,756	1.23	1.07–1.42	1.23	1.06–1.42	1.19	0.99–1.43
≥30	1026	72	10,947	1.27	0.98–1.63	1.22	0.94–1.58	1.14	0.79–1.64
Average BMI from BMI_67_ to baseline HUNT-3									
<18.5	65	1	742	0.46	0.06–3.25	0.41	0.06–2.95	0.41	0.06–2.96
18.5–<25	8477	401	93,931	1	(Ref)	1	(Ref)	1	(Ref)
25–29.9	4865	393	50,683	1.30	1.13–1.50	1.29	1.11–1.48	1.29	1.08–1.52
≥30	732	61	7487	1.33	1.01–1.75	1.27	0.96–1.67	1.27	0.90–1.79

N: number of participants within each category; HR: hazard ratio; CI: confidence interval. § Model 1: adjusted for age at baseline (continuous) and sex. ‖ Model 2: adjusted as model 1 and additional for smoking status (never, former, current), education (lower secondary education, upper secondary education, tertiary education), marital status (unmarried, married/cohabitant, widowed/divorced/separated), physical activity (inactive, active), alcohol consumption (abstain, light drinkers, moderate drinkers, heavy drinkers) and chronic diseases (number of chronic diseases). # Adjustments as in Model 2 and additionally for the most recent BMI (BMI from HUNT-3).

## Data Availability

Data from the HUNT study used in this research project is available upon reasonable request to the HUNT data access committee (hunt@medisin.ntnu.no).

## Supplementary Materials

The following supporting information can be downloaded at: https://www.mdpi.com/article/10.3390/nu15051232/s1, Figure S1: Flowchart of the population. Exclusion criteria due to missing information were not mutually exclusive. HUNT Trøndelag Health Study; BMI Body mass index; File S1: Supplementary material-BMI trajectory modeling [19,39,40,41,42,43]; Table S1: Hazard Ratios (HRs) of Ischemic Stroke, by Categories of Average Body Mass Index to the end of Follow-up with Repeated Updated Exposure and by Categories of Average Body Mass Index Until the HUNT-3 Measurement (BMI_07_). Table S2: Characteristics of Participants of the Three Group-Based Trajectories of repeated BMI Measurement. Table S3: Hazard Ratios (HRs) of Ischemic Stroke, by Categories of Average Body Mass Index to the end of follow-up with repeated updated exposure, stratified for sex, age at baseline (<65 years and ≥65 years) and smoking status. Table S4: Hazard Ratios (HRs) of Ischemic Stroke, by Categories of Body Mass Index, for BMI Groups Separately for each Examination (Tbc-screening, HUNT1, 2, 3) and by Categories of Body Mass Index Change in the Whole, Early, Middle and Late Period. Table S5: GBTM-Table 1. Bayesian information for body mass index (BMI) group-based trajectory modeling (GBTM) group according to number of groups and trajectory shapes; Table S6: GBTM-Table 2. Average posterior probability (AvePP) value and odds of correct classification for BMI GBTM groups; Table S7: GBTM-Table 3. Body mass index trajectory groups’ estimated proba-bility and the proportion of study members classified to each group according to the maximum posterior probability assignment rule.
