# Supplementary material for: Body Mass Index Measured Repeatedly over 42 Years as a Risk Factor for Ischemic Stroke: The HUNT Study
Source: Nutrients. 2023 Feb 28;15(5):1232. doi: 10.3390/nu15051232 (PMC10005195; doi:10.3390/nu15051232)
Supplement: Supplementary file 1 [file nutrients-15-01232-s001.zip › nutrients-2173852-supplementary.pdf]

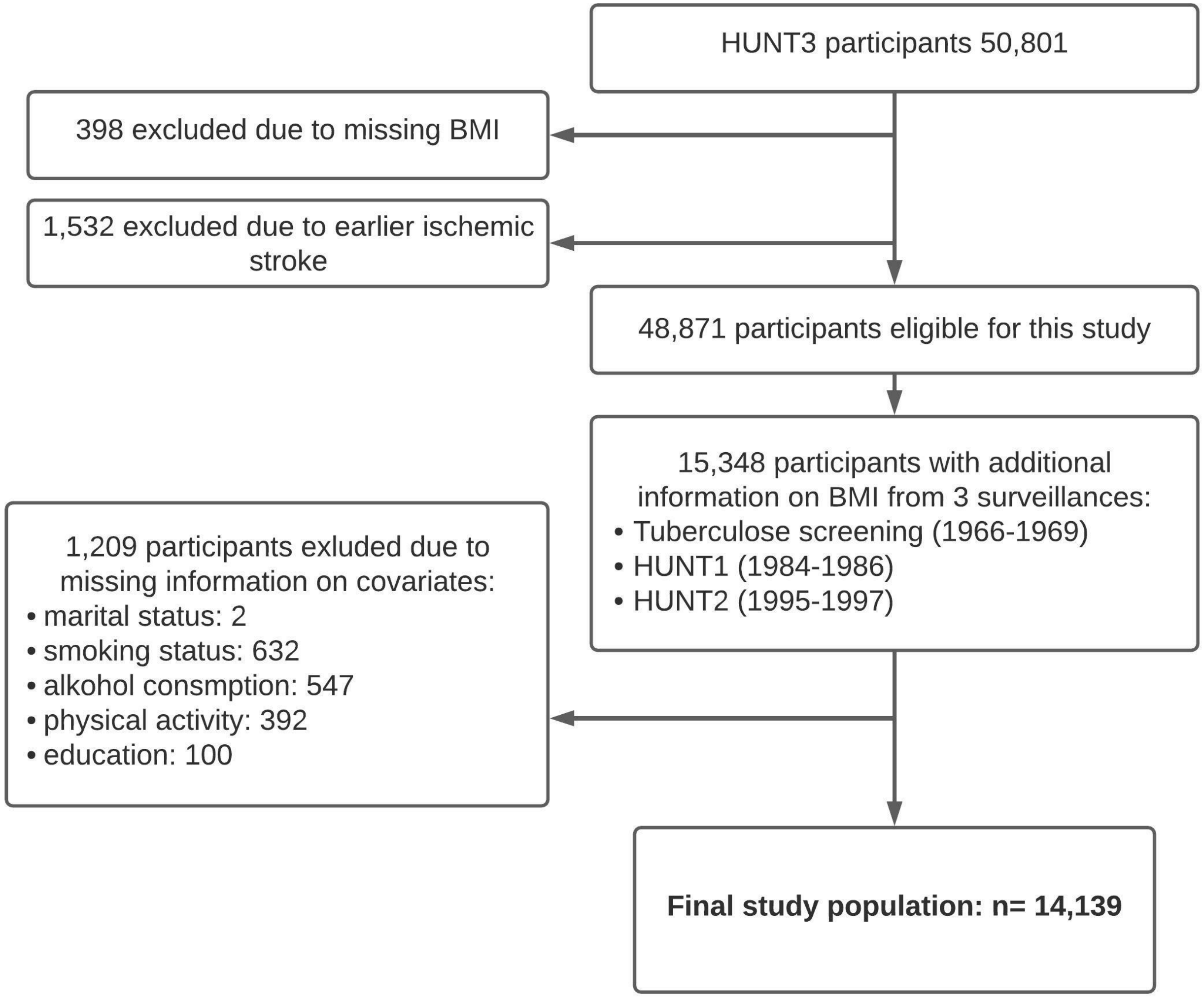
HUNT3 participants 50,801
398 excluded due to missing BMI
1,532 excluded due to earlier ischemic stroke
48,871 participants eligible for this study
15,348 participants with additional information on BMI from 3 surveillances:
• Tuberculose screening (1966-1969)
• HUNT1 (1984-1986)
• HUNT2 (1995-1997)
1,209 participants exluded due to missing information on covariates:
• marital status: 2
• smoking status: 632
• alkohol consmption: 547
• physical activity: 392
• education: 100
Final study population: n= 14,139

Figure S1: Flowchart of the population. Exclusion criteria due to missing information were not mutually exclusive. HUNT Trøndelag Health Study; BMI Body mass index

Table S1. Hazard Ratios (HRs) of Ischemic Stroke, by Categories of Average Body Mass Index to the end of Follow-up with Repeated Updated Exposure and by Categories of Average Body Mass Index Until the HUNT-3 Measurement (BMI_{07}).

Mean BMI (kg/m²)	N 14 139	Events 856	Person years 152 843	HR[§]	95% CI	HR[#]	95% CI
Average BMI from BMI_{67} to the end of follow up with repeated updated exposure							
<18.5	36	1	405	0.55	0.08-3.90	0.58	0.08-4.12
18.5-<25	7 182	363	78 735	1	(Ref)	1	(Ref)
25-29.9	5 895	420	62 756	1.23	1.06-1.42	1.16	1.00-1.35
≥30	1 026	72	10 947	1.22	0.94-1.58	1.06	0.80-1.39
Mean BMI (kg/m²)	N	Events	Person years	HR[§]	95% CI	HR[#]	95% CI
Average BMI from BMI_{67} to baseline HUNT-3							
<18.5	65	1	742	0.41	0.06-2.95	0.43	0.06-3.08
18.5-<25	8 477	401	93 931	1	(Ref)	1	(Ref)
25-29.9	4 865	393	50 683	1.29	1.11-1.48	1.23	1.06-1.42
≥30	732	61	7 487	1.27	0.96-1.67	1.10	0.82-1.47

N: number of participants within each category; HR: hazard ratio; CI: confidence interval.

§ Adjusted for age at baseline (continuous), sex, smoking status (never, former, current), education (lower secondary education, upper secondary education, tertiary education), marital status (unmarried, married/cohabitant, widowed/divorced/separated), physical activity (inactive, active), alcohol consumption (abstain, light drinkers, moderate drinkers, heavy drinkers) and chronic diseases (number of chronic diseases). Same as Model 2 in Table 2.

# Adjustments as above and additionally for hypertension (Yes/No) defined by increased blood pressure or use of antihypertensive medication, diabetes (Yes/NO) defined by increased non fastening glucose or use of diabetes medication, triglycerides as continuous variable and HDL pathological reduced (Yes/No).

Table S2. Characteristics of Participants of the Three Group Based Trajectories of repeated BMI Measurement.

	Constantly Normal Weight Group n= 6 900	Developing Overweight Group n= 6 149	Developing Obesity Group n= 1 090
Age at HUNT3 (years)	65.0 (9.4)	67.4 (9.2)	67.8 (8.7)
Women, n (%)	4 077 (59.1)	3 027 (49.2)	734 (67.3)
BMI_{67}(kg/m^2)	21.5 (2.2)	24.2 (2.5)	28.2 (3.7)
BMI_{85}(kg/m^2)	22.3 (1.8)	26.1 (1.9)	31.5 (3.2)
BMI_{96}(kg/m^2)	23.9 (1.9)	28.4 (1.9)	34.7 (3.2)
BMI_{07}(kg/m^2)	24.6 (2.3)	29.5 (2.4)	35.9 (3.7)
SBP (mmHg)	134.9 (19.5)	139.9 (19.1)	140.9 (18.9)
DBP (mmHg)	74.2 (11.2)	76.5 (11.5)	74.9 (11.0)
Hypertension, n (%)	4 566 (66.2)	4 985 (81.1)	999 (91.7)
Diabetes mellitus, n (%)	220 (3.2)	571 (9.3)	227 (20.8)
Total cholesterol (mmol/L)	5.9 (1.1)	5.7 (1.1)	5.5 (1.1)
HDL cholesterol (mmol/L)	1.5 (0.4)	1.3 (0.9)	1.2 (0.3)
Triglycerides (mmol/L)	1.5 (0.8)	1.9 (1.0)	2.1 (1.2)
Smoking status			
Never, n (%)	2 623 (38.0)	2 415 (39.3)	490 (45.0)
Former, n (%)	2 647 (38.4)	2 602 (42.3)	432 (39.6)
Occasionally, n (%)	355 (5.1)	343 (5.6)	50 (4.6)
Current, n (%)	1 275 (18.5)	789 (12.8)	118 (10.8)
Alcohol consumption			
Abstainers, n (%)	1 509 (21.9)	1 624 (26.4)	393 (36.1)
Light drinkers, n (%)	3 853 (55.9)	3 404 (55.4)	553 (50.7)
Moderate drinkers, n (%)	1 424 (20.6)	1 031 (16.8)	134 (12.3)
Heavy drinkers, n (%)	114 (1.7)	90 (1.5)	10 (0.9)
Education			
Lower secondary, n (%)	2 766 (40.1)	2 848 (46.3)	628 (57.6)
Upper secondary, n (%)	2 863 (41.5)	2 423 (39.4)	351 (32.2)
Tertiary, n (%)	1 271 (18.4)	878 (14.3)	111 (10.2)
Physical inactivity			
Inactive, n (%)	1 140 (16.5)	1 342 (21.8)	359 (32.8)
Active, n (%)	5 760 (83.5)	4 807 (78.2)	732 (67.2)
Marital Status			
Single, n (%)	407 (5.9)	302 (4.9)	73 (6.7)
Married, Cohabitant, n (%)	4 879 (70.7)	4 271 (71.1)	712 (65.3)
Widow, Divorced, Separated, n (%)	1 614 (23.4)	1 476 (24.0)	305 (28.0)

Values are presented as mean ± standard deviation or number (percentages). BMI body mass index, SBP systolic blood pressure, DBP diastolic blood pressure, HDL high-density lipoprotein. Not indicated number of missing.

Table S3. Hazard Ratios (HRs) of Ischemic Stroke, by Categories of Average Body Mass Index to the End of Follow-up with Repeated Updated Exposure, Stratified for Sex, Age at Baseline (<65 years and ≥65 years) and Smoking Status

	Women					Men					
BMI (kg/m²)	N 7,838	Events 417	Person years 86,404	HR	95% CI	N 6,301	Events 439	Person years 66,439	HR	96% CI	p for interaction
<18.5	28	1	410	0.81	0.11-5.76	8	0	108	-		-
18.5-<25	4,215	194	50,124	1	(Ref)	2,960	169	34,449	1	(Ref)	(Ref)
25-29.9	2,896	173	29,194	1.07	0.86-1.32	3,005	247	29,031	1.37	1.12-1.66	0.209
≥30	699	49	6,676	1.11	0.80-1.54	328	23	2.852	1.26	0.81-1.96	0.899

	<65 years					≥65 years					
BMI (kg/m²)	N 6,972	Events 205	Person years 81,024	HR	95% CI	N 7,167	Events 651	Person years 71,816	HR	96% CI	
<18.5	25	1	393	1.23	0.17-8.83	11	0	125	-		-
18.5-<25	4,118	108	51,671	1	(Ref)	3,057	255	32,902	1	(Ref)	(Ref)
25-29.9	2,465	86	25,537	1.40	1.05-1.87	3,436	334	32,687	1.26	1.07-1.48	0.280
≥30	364	10	3,427	1.22	0.63-2.35	663	62	6,101	1.24	0.93-1.64	0.885

	Former or Current Smoker					Never Smoker					
BMI (kg/m²)	N 8,611	Events 536	Person years 92,095	HR	95% CI	N 5,528	Events 320	Person years 60,748	HR	96% CI	
<18.5	25	1	352	0.76	0.11-5.43	11	0	165	-	-	-
18.5-<25	4,487	240	52,232	1	(Ref)	2,688	123	32,341	1	(Ref)	(Ref)
25-29.9	3,559	261	34,523	1.19	0.99-1.42	2,342	159	23,702	1.25	0.98-1.59	0.492

≥30	540	34	4,988	1.09	0.76-1.57	487	38	4,540	1.29	0.89-1.88	0.309

N: total numbers within each category; HR: hazard ratio; CI: confidence interval.

Adjusted for sex, age at baseline, smoking status (never, former, current), education (lower secondary education, upper secondary education, tertiary education), marital status (unmarried, married/cohabitant, widowed/divorced/separated), physical activity (inactive, active), alcohol consumption (abstain, light drinkers, moderate drinkers, heavy drinkers) and chronic diseases (number of chronic diseases) (as in Model 2, Table 2).

Table S4: Hazard Ratios (HRs) of Ischemic Stroke, by Categories of Body Mass Index, for BMI Groups Separately for each Examination (Tbc-screening, HUNT1, 2, 3) and by Categories of Body Mass Index Change in the Whole, Early, Middle and Late Period.

	Tbc screening			HUNT1			HUNT2			HUNT3		
BMI (kg/m^2)	N (Events)	HR	95%CI	N (Events)	HR	95%CI	N (Events)	HR	95%CI	N (Events)	HR	95%CI
<18.5	243 (9)	1.0	0.52-1.96	144 (1)	0.21	0.03-1.48	47 (4)	1.53	0.57-4.11	75 (4)	0.80	0.30-2.17
18.5-<25	10 257 (526)	1	(Ref.)	8 157 (391)	1	(Ref.)	4 799 (241)	1	(Ref.)	3 752 (213)	1	(Ref.)
25-29.9	3 222 (281)	1.27	1.09-1.47	4 905 (391)	1.31	1.13-1.51	6 922 (436)	1.10	0.94-1.29	6 768 (401)	1.01	0.85-1.19
≥30	417 (40)	1.33	0.96-1.84	933 (73)	1.29	1.00-1.66	2 371 (175)	1.29	1.05-1.57	3 544 (238)	1.17	0.97-1.42

	Whole period (1967-2007)			Early period (1967-1985)			Middle period (1985-1996)			Late period (1996-2007)		
BMI change in kg/m^2	N(Events)	HR	95%CI	N(Events)	HR	95%CI	N(Events)	HR	95%CI	N(Events)	HR	95%CI
<-2.5	28(3)	1,47	0.47-4.63	528(25)	0.77	0.52-1.16	150(10)	0.97	0.52-1.83	789(73)	1.34	1.03-1.75
≥-2.5 to <2.5	11 111(727)	1	(Ref.)	9 354(616)	1	(Ref.)	8 769(559)	1	(Ref.)	10 459(642)	1	(Ref.)
≥2.5	3 000(126)	1.02	0.82-1.26	4 257(215)	1.03	0.88-1.21	5 220(287)	1.16	0.98-1.37	2 891(141)	0.96	0.78-1.19

N: total number within each category; HR: hazard ratio; CI: confidence interval.

Adjusted for sex, age at baseline (continuous), smoking status (never, former, current), education (lower secondary education, upper secondary education, tertiary education), marital status (unmarried, married/cohabitant, widowed/divorced/separated), physical activity (inactive, moderate, vigorous), alcohol consumption (abstainer, light drinker, moderate drinker, heavy drinker) and chronic diseases (number of chronic diseases) (model 2).

File S1. Supplementary material-BMI trajectory modelling

Group based trajectory modelling (GBTM)

Group based trajectory modelling (GBTM)[39] is a specialized tool for analyzing developmental trajectories over time.[40, 41] This method has shown to be suitable for identifying underlying longitudinal trajectories.[42]

We used the The Traj Stata Plugin from Carnegie Mellon University (www.andrew.cmu.edu/user/bjones/traj) to estimate group-based trajectory models.[19] This Traj Plugin allows calculation of the probability of group membership, predict the trajectory for each group and the posterior probabilities of group membership. To model the BMI trajectories we used the censored normal distribution which is designed to analysis repeatedly measured continuous variables.[43]

For model selection we used the Bayesian information criteria.[19] In addition was the model chosen by iterative estimation[41] of the parsimony of the model which fitted the data well, adequate correspondence between the proportion of sample numbers and estimated probability in each group based on the maximum posterior probability assignment rule. The odds of correct classification based on the posterior probabilities of group membership >5 for each group (the shape/order of each trajectory group) and an average posterior probability value >0.7 for each group. In addition was it necessary that the identified trajectories fulfilled scientific evident biological plausibility.

Procedure of identifying BMI trajectories by using group-based trajectory modelling (GBTM)

We started with initially 2 groups, testing different orders (i.e., zero-order, linear and quadratic) for the trajectory shapes and increased the number of groups up to 5 until the best fitting model was established.

The 3-group model with one quadratic and two linear trajectories (2 1 1) was chosen based on the model selection criterion. The model had an adequate proportion and sample number in each group: "normal weight" 48.8%, "developing overweight" 43.4%, and "developing obese" 7.8% (Figure S3). The average posterior probability (AvePP) value was 0.93 or more for each group; larger than the recommended minimum AvePP value of 0.70 (GBTM-Table 2). The odds of correct classification were over 5.0 for all three groups, indicating the model had good assignment accuracy (GBTM-Table 2). And there was close correspondence between estimated probability and the proportion of study members assigned to it in each group (GBTM-Table 3).

GBTM-Table 1. Bayesian information for body mass index (BMI) group-based trajectory modeling (GBTM) group according to number of groups and trajectory shapes			
Number of groups	Trajectory shape[1] (percent participants in each group)	BIC[2](n=56,556)	BIC[3](n=14,139)
2	00* (77.9, 22.1)	-152,929	-152,926
2	01* (59.9, 40.1)	-147,407	-147,404
2	02* (55.5, 44.5)	-147,371	-147,367
2	11* (73.6, 26.4)	-143,074	-143,070
2	12 (71.6, 28.4)	-143,882	-143,877
2	22 (63.3, 34.7)	-147,328	-147,322
3	000 (27.9, 48.5, 23.6)	-153,403	-153,399
3	001 (16.8, 54.2, 29.0)	-146,743	-146,739
3	010 (33.5, 50.4, 16.0)	-150,659	-150,655
3	011 (25.3, 60.7,14.0)	-140,127	-140,122
3	012(32.5,52.5,15.0)	-144,961	-144,955
3	002(25.2,56.8,18.0)	-148,663	-148,657
3	111 (55.1, 23.1, 6.7)	-140,224	-140,184
3	112 (55.2, 33.9, 8.9)	-144,335	-144,328
3	121 (57.2, 33.9, 8.9)	-143,200	-143,193
3	122 (39.3, 49.7, 11.0)	-143,507	-143,500
3	210 (48.8, 45.3, 5.9)	-144,347	-144,341
3	211 (48.8, 43.4, 7.8)	-139,963	-139,956
3	212 (49.0, 35.8, 15.2)	-152,312	-152,305
3	221 (46.9, 41.6, 11.5)	-147,422	-147,415
3	222 (38.3, 50.5, 11.2)	-142,888	-142,834
4	0000* (38.2, 47.8, 12.3, 1.7)	-150,397	-150,391
4	1111* (48.0, 27.7, 20.3, 4.0)	-136,252	-136,243
4	1112* (47.2, 39.8, 11.9, 1.1)	-140,223	-140,214
4	1121* (32.2, 15.5, 49.6, 2.7)	-136,831	-136,822
4	1122* (25.3, 48.8, 21.7, 4.2)	-138,425	-138,416
4	1211* (39.8, 32.8, 18.3, 3.1)	-139,542	-139538
4	1221* (28.1 ,47.8, 20.1, 4.0)	-136,245	-136,235
4	1212* (40.2, 37.0, 15.6, 7.2)	-141,631	-141,621
4	1222*(36.4, 32.8, 21.7, 9.0)	-151,845	-151,835
4	2222(31.1, 29.9, 25.2, 13.8)	-151,006	-150,934
5	00000* (24.5,48.7,21.0,5.2,0.6)	-150,184	-150,177

5	11111* (18.8,28.0,43.0,8.6,1.5)	-135,116	-135,106

[1] Trajectory shape; 0=zero-order; 1= linear; 2=quadratic.
[2] BIC = Bayesian information criterion (for total number of participants)
[3] BIC = Bayesian information criterion (for total number of observations)
* (One or more of the groups have a very small proportion of observations, i.e., less than 5%;) Variance matrix is nonsymmetric or highly singular

GBTM-Table 2. Average posterior probability (AvePP) value and odds of correct classification for BMI GBTM groups

	BMI GBTM groups		
	Normal weight	Developing Overweight	Developing Obese
Average posterior probability value	0.95	0.93	0.95
Odds of correct classification	19.70	18.01	243.37

GBTM-Table 3. Body mass index trajectory groups' estimated probability and the proportion of study members classified to each group according to the maximum posterior probability assignment rule

	BMI GBTM groups		
	Normal weight	Developing Overweight	Developing Obese
Estimated group probability	48.8	43.5	7.7
Proportion assigned to group according to the maximum posterior probability assignment rule	48.8	43.4	7.8
